# The role of A-kinase anchoring protein 95-like protein in annealing of tRNA^Lys3^ to HIV-1 RNA

**DOI:** 10.1186/1742-4690-11-58

**Published:** 2014-07-17

**Authors:** Li Xing, Xia Zhao, Fei Guo, Lawrence Kleiman

**Affiliations:** Lady Davis Institute for Medical Research and McGill AIDS Centre, Jewish General Hospital, Montreal, QC Canada; Department of Medicine, McGill University, Montreal, QC Canada; State Key Laboratory for Molecular Virology and Genetic Engineering, Institute of Pathogen Biology, Chinese Academy of Medical Sciences, Beijing, 100730 People’s Republic of China

**Keywords:** HIV-1, RNA helicase A, HAP95, tRNA^Lys3^ annealing

## Abstract

**Background:**

RNA helicase A (RHA), a DExH box protein, promotes annealing of tRNA^Lys3^, a primer for reverse transcription, to HIV-1 RNA and assembles into virus particles. A-kinase anchoring protein 95-like protein (HAP95) is a binding partner of RHA. The role of HAP95 in the annealing of tRNA^Lys3^ was examined in this study.

**Results:**

HAP95 associates with the reverse transcriptase region of Pol protein of HIV-1. Decreasing endogenous HAP95 in HIV-1-producing 293T cells by siRNA reduces the amount of tRNA^Lys3^ annealed on viral RNA. This defect was further deteriorated by knockdown of RHA in the same cells, suggesting a cooperative effect between these two proteins. Biochemical assay *in vitro* using purified GST-tagged HAP95 shows that HAP95 may inhibit the activity of RHA.

**Conclusion:**

The results support a hypothesis that HAP95 may transiently block RHA’s activity to protect the annealed tRNA^Lys3^ on viral RNA in the cells from removing by RHA during the packaging of RHA into virus particles, thus facilitating the annealing of tRNA^Lys3^ to HIV-1 RNA.

## Background

Reverse transcription is a signature step of retrovirus replication that produces minus strand strong stop cDNA (ssscDNA) upon infection of the new cells. Reverse transcriptase (RT) is the major enzyme responsible for reverse transcription and was released from Pol by proteolytic cleavage of a polyprotein precursor Gag-Pol during the budding of virus particles [1]. HIV-1, a member of lentivirus family of retroviridae, selectively packages tRNA^Lys3^ into virus particle and anneals tRNA^Lys3^ to viral genomic RNA as a primer in reverse transcription [2]. The annealing of tRNA^Lys3^ consists of the hybridization of the 3’ terminal 18 nucleotides (nts) of tRNA^Lys3^ to a complementary sequence of 18 nts termed the primer binding site (PBS) of HIV-1 genomic RNA and several other distinct base-paring interactions between tRNA^Lys3^ and viral RNA sequences upstream of PBS including the tRNA^Lys3^ anticodon complimentary to a small single-stranded A-rich loop [3], the 5’ part of the TYC arm complementary to 8 nts primer activation site (PAS) [4], and 6 nts sequence containing part of variable loop complementary to a cytidine-rich region upstream of viral A-rich loop [5].

A number of viral and cellular protein factors participate in reverse transcription-associated activities of HIV-1 [6]. For instance, Gag plays crucial roles in a variety of reverse transcription-related activities including the incorporation of viral genomic RNA into virions [7], the dimerization of viral genomic RNA [8], and the tRNA^Lys3^ annealing to viral genomic RNA [9]. The annealing of tRNA^Lys3^ to viral genomic RNA was initially mediated by Gag [10, 11] and then fine-tuned by nucleocapsid (NC) protein, a proteolytic derivative of Gag during budding of virus particles, to render the annealed tRNA^Lys3^ an increased capability as a reverse transcription primer [12, 13].

Human RNA helicase A (RHA), also known as DHX9, is a member of DExD/H box protein family and has also been shown involved in reverse transcription-related activity of HIV-1. For example, RHA has been shown to promote the annealing of tRNA^Lys3^ to viral RNA [14] and is packaged into HIV-1 particles [15]. Knockdown of endogenous RHA by RNAi technique in virus-producing cells results in reduced annealing of tRNA^Lys3^ to viral RNA in progeny virions.

A-kinase anchoring protein 95-like protein, also named HAP95, HA95, or NAKAP95, is a product of gene duplication of A-kinase anchoring protein 95 (AKAP95) [16, 17] and regulates a variety of physical or pathological processes including chromatin condensation, and initiation of DNA replication [16, 18], pre-mRNA splicing [19], pathogenesis of Huntington’s disease (HD) [20], and oncogenesis [21]. HAP95 was identified as a cellular binding partner of RHA [22] and can synergize with RHA to activate the cis-acting constitutive transport element (CTE)-mediated gene expression of type D retroviruses [23]. In analysis of HIV-1 Pol protein complex, we found that HAP95 associates with RT part of Pol. Thus, we wonder whether HAP95 executes a role in reverse transcription-related activities of HIV-1. We found that knockdown of HAP95 in virus-producing 293T cells does not affect the production of HIV-1 particle nor does the viral RNA packaging, but reduces the annealing of tRNA^Lys3^ to viral RNA. This defect in tRNA^Lys3^ annealing was deteriorated by knockdown of RHA in the same cells. However, *in vitro* tRNA^Lys3^ annealing assay using purified GST-tagged HAP95 revealed that HAP95 inhibits the activity of RHA. These seemingly contradictory observations suggest that HAP95 may work as a negative regulator for the activity of RHA in promotion of tRNA^Lys3^ annealing.

## Results

### HAP95, an RHA binding protein, associates with reverse transcriptase region of HIV-1 Pol protein and is incorporated into HIV-1 particles upon overexpression

**Figure 1:**
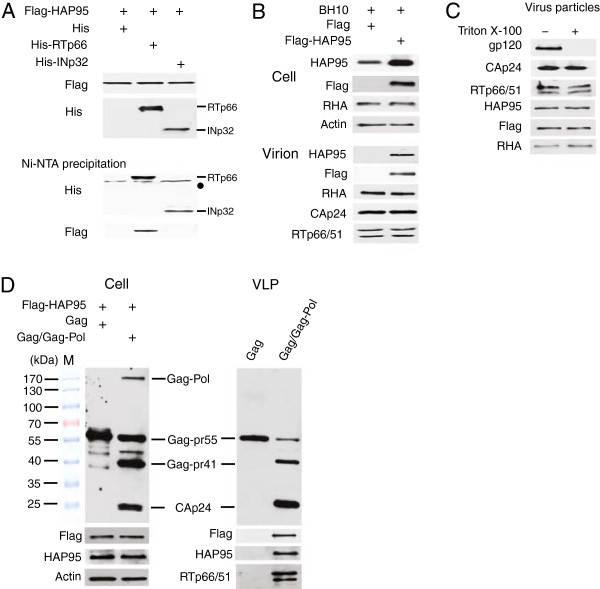
**Incorporation of HAP95 into HIV**-**1 particles upon overexpression. (A)** Co-precipitation of HAP95 with His-tagged RTp66. 293 T cells were cotransfected with DNAs encoding for Flag-HAP95 and either His-tagged RTp66 or His-tagged INp32, or His tag. Upper panels show Western blots of cell lysates probed with either anti-Flag or anti-His. The cell lysates were then incubated and precipitated with Ni-NTA agarose. The lower panels show the Western blots of the precipitates probed with either anti-His or anti-Flag. •, non-specific protein bands. The His-tag peptide alone was not detected in these Western blots. **(B)** Association of HAP95 with purified HIV-1 particles. 293 T cells were cotransfected with DNAs encoding for HIV-1 BH10 and either Flag-HAP95 or Flag tag. Upper panels show the Western blots of cell lysates probed with anti-HAP95, anti-Flag, anti-RHA, and anti-β-actin. Lower panels show the Western blots of purified HIV-1 particles probed with anti-HAP95, anti-Flag, anti-RHA, anti-p24, and anti-RT. **(C)** HIV-1 particles containing Flag-HAP95 were treated with Triton X-100. The viral cores were isolated and analyzed by Western blotting using anti-gp120, anti-p24, anti-RT, anti-HAP95, anti-Flag, and anti-RHA. **(D)** Incorporation of HAP95 into VLP. 293T cells were cotransfected with DNAs encoding for Flag-HAP95 and either BH10 Gag or BH10 Gag/Gag-Pol. VLPs were purified by step sucrose centrifugation at 48 hours posttransfection. Cell lysates and VLPs were analyzed by Western blotting using anti-p24, anti-Flag, anti-HAP95, anti-β-actin, or anti-RT. M, protein size marker shown in kDa.

The TAP (tandem affinity protein purification system) method has been widely used to study the protein complex in the cell. Roy et al. used this method to study the cellular proteins that associate with HIV-1 Gag [15] and identified RHA that is incorporated into HIV-1 particles and promotes the synthesis of viral cDNA upon viral infection of the new cells. Pol consists of protease, RT and integrase (INp32). Both Pol and its processed product RT play important roles in the selection of tRNA^Lys3^ as a primer for HIV-1 reverse transcription [24, 25]. In this report, we used TAP to identify cellular proteins binding to the Pol part of HIV-1 Gag-Pol polyprotein. A plasmid pcDNA3-N-TAP-hPol was constructed and the Pol protein encoded by this plasmid has amino acid sequence identical to that of HIV-1 NL4-3 Pol, but the mRNA sequence has their codons optimized for mammalian cell codon usage. 293T cells were stably transfected with pcDNA3-N-TAP-hPol. TAP-Pol associated proteins were isolated by two-step affinity chromatography, resolved in 1D sodium dodecyl sulfate (SDS)-polyacrylamide gels (PAGE). The proteins in different bands were identified by mass spectrometry analysis. One of the proteins associated with Pol was found to be HAP95, an RHA binding protein [22, 23]. We then determined whether HAP95 coprecipitates with RTp66 or INp32 by cotransfecting 293 T cells with DNAs expressing Flag-HAP95 and either His-RTp66 or His-INp32. Figure 1A show the ability of Flag-HAP95 to be coprecipitated with RTp66 from cellular lysates. Western blots of cellular lysates probed with anti-Flag or anti-His (upper panel) show the expression of Flag-HAP95 or His-RTp66 and His-INp32 in the cells. Western blots of the precipitates probed with either anti-His or anti-Flag (lower panel) show that Flag-HAP95 coprecipitates with RTp66, but not with INp32.One implication of Pol-HAP95 association is the incorporation of HAP95 into virus particles. To test this hypothesis, 293 T cells were cotransfected with SVC21.BH10 containing full-length HIV-1 BH10 proviral DNA and a plasmid expressing Flag-HAP95 or only Flag tag. The virus particles thus produced were purified through step sucrose centrifugation and analyzed by Western blotting using anti-Flag or anti-HAP95. HAP95 was not detected in the virus particles until it was over-expressed (Figure 1B). This observation was further confirmed by the analysis of core of HIV-1 particles (Figure 1C). The purified virus particles containing Flag-HAP95 were first treated with Triton X-100 to deplete the virus envelope. After extensive washing, the viral core pellet was assessed for the presence of viral envelope protein (gp120) and retention of HAP95. The results of Western blotting show that the envelope protein was removed by treatment with Triton X-100, and that the RT, CAp24, and HAP95 were recovered with the viral core pellets, indicating that HAP95 was packaged in the virus particles. As expected, RHA was readily detected in the cells and in the HIV-1 particles (Figure 1B and C). Moreover, the level of RHA in virus particles was not changed obviously by the incorporation of HAP95 into virus particles.In order to test the role of Pol in the incorporation of exogenous HAP95 into HIV-1 particles, we analyzed virus-like particles (VLPs) of Gag or of Gag/Gag-Pol. These two kinds of VLPs were produced in 293T cells expressing Flag-tagged HAP95 (Figure 1D). Gag VLP contains unprocessed Gag-pr55. Gag/Gag-Pol VLP mainly contains the processed Gag and Pol. The expression of functional Pol was demonstrated by the detection of unprocessed Gag-Pol in Gag/Gag-Pol VLP-producing 293T cells (left panel) and processed products of Pol, RTp66/51 in purified Gag/Gag-Pol VLP (right panel) using anti-p24 and anti-RT respectively. Flag-tagged HAP95 was detected in Gag/Gag-Pol VLP instead of Gag VLP, suggesting a role of Pol in the incorporation of overexpressed HAP95 into virus particles.

### HAP95 is not required for production of HIV-1 particle, but is important for infectivity of progeny virion

**Figure 2:**
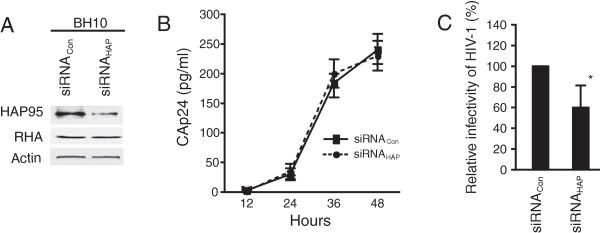
**Decreased cellular HAP95 does not affect the production of HIV-1 particles, but reduces the infectivity of virus particles.** 293 T cells were treated with siRNA_Con_ or siRNA_HAP_, and 16 hours later, were transfected with DNA encoding for HIV-1 BH10. **(A)** Cell lysates were prepared at 24 hours posttransfection of DNA and analyzed by Western blotting using anti-HAP95, anti-RHA, or anti-β-actin. **(B)** The extracellular culture medium was analyzed by CAp24 ELISA assay at the indicated time points posttransfection of DNA to determine the amount of virus particles. **(C)** Viral infectivity assay. HIV-1 particles were purified at 48 hours posttransfection of DNA and the single-round viral infectivity was measured by challenging TMZ-b1 cell with purified viruses corresponding to 5 ng of CAp24. The induced luciferase activity was determined at 24 hours later and the results were normalized to that of virions produced from siRNA_Con_-treated cells. Shown are the mean values ± standard deviation (SD) of results of 3 experiments. *, *P* <0.05.

To study the role of HAP95, we analyzed the production of HIV-1 particles under condition of reduced endogenous HAP95. 293T cells were first treated with either siRNA_Con_ or siRNA_HAP_, and 16 hours later, were transfected with SVC21.BH10 DNA coding for HIV-1 BH10. Western blots of cell lysates probed with anti-HAP95 or anti-RHA shows that HAP95 was effectively reduced by siRNA_HAP_, but the level of RHA was not changed (Figure 2A). The production of extracellular HIV-1 particles was determined by measuring the amount of CAp24 at the indicated time points posttransfection. The results show that knockdown of HAP95 did not affect the yields of virus particles over the time course (Figure 2B). However, in single-cycle infectivity assay, we found that the reduced HAP95 affected the infectivity of produced virus particles. Thus, challenging TZM-bl indicator cells with viruses produced in the cells treated with siRNA_HAP_ resulted in reduction in the induced luciferase activity in the cell lysates (Figure 2C).

### HAP95 is not required for viral RNA packaging, but is important for the annealing of tRNA^Lys3^ to HIV-1 RNA

**Figure 3:**
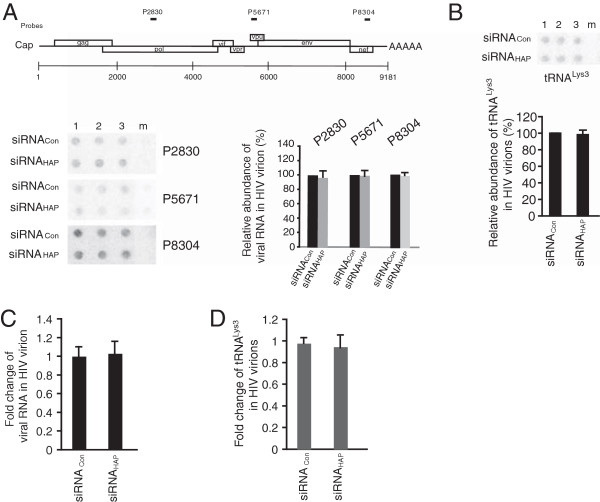
**Decreased cellular HAP95 does not affect the incorporation of viral RNA and tRNA** ^**Lys3**^ **into HIV-1 particles.** Total viral RNA was isolated from equal amounts of purified HIV-1 particles. The abundance of viral RNA or tRNA^Lys3^ was determined by hybridizing dot blots of total viral RNA with ^32^P-labeled DNA probes specific for HIV-1 RNA or tRNA^Lys3^ and quantitating radioactive signals using a PhosphorImager instrument. The results were normalized to that of virions produced from siRNA_Con_-treated cells and presented in percentage. **(A)** Upper panel: Diagram of HIV-1 genomic organization and the positions of 3 DNA probes used in dot blot hybridization. Numbers indicate the nucleotide positions relative to the start site (+1) of transcription of HIV-1 genomic RNA. Lower panel: A representative of dot blots probed with 3 DNA probes in triplicate (left part, 1, 2, and 3) and a graph showing the relative abundance of viral genomic RNA in HIV-1 virions (right part). m, samples obtained from mock-transfected cells. **(B)** A representative of dot blots probed with tRNA^Lys3^ probe in triplicate (upper panel, 1, 2, and 3) and a graph showing the relative abundance of tRNA^Lys3^ in HIV-1 virions (lower panel). **(C, D)** Quantitation of viral RNA and tRNA^Lys3^ in HIV-1 particles by real-time RT-PCR. Shown are the averages of fold change ± SD of 3 experiments.

We then analyzed the incorporation of viral RNA and tRNA^Lys3^ in the virus particles by performing dot blot hybridization and real-time RT-PCR. The total viral RNA was isolated from equal amounts (about 500 ng of CAp24) of each virus preparation. In dot blot hybridization, the RNA was blotted in triplicate and then was hybridized with ^32^P-labeled DNA probes targeting three different regions of HIV-1 RNA genome or with ^32^P-labeled DNA probe specific to tRNA^Lys3^. The samples obtained from mock- transfected cells were used as a negative control. The results were analyzed using a PhosphorImager instrument. Neither dot blot hybridization nor real-time RT-PCR detected the obvious change in viral RNA incorporation as a result of reduction in HAP95 content in virus-producing cells (Figure 3A and C). The packaging of tRNA^Lys3^ remains unchanged as well (Figure 3B and D).

To determine whether the reduced viral infectivity reflects an alteration in the annealing of tRNA^Lys3^ to viral RNA, we examined the ability of tRNA^Lys3^ to be extended in an *in vitro* reverse transcription system, using deproteinized total viral RNA as the source of primer tRNA^Lys3^ annealed *in vivo* to viral genomic RNA. The relative amount of tRNA^Lys3^ annealed to viral RNA is determined by +6 nt extension under conditions in which all other reagents in the reaction are in excess, so that the amount of tRNA^Lys3^ extended is proportional to the amount of total viral RNA in the reaction mixture [10]. We have also examined incorporation of the first dNTP, dCTP, into tRNA^Lys3^ annealed to viral RNA (+1 nt extension).

**Figure 4:**
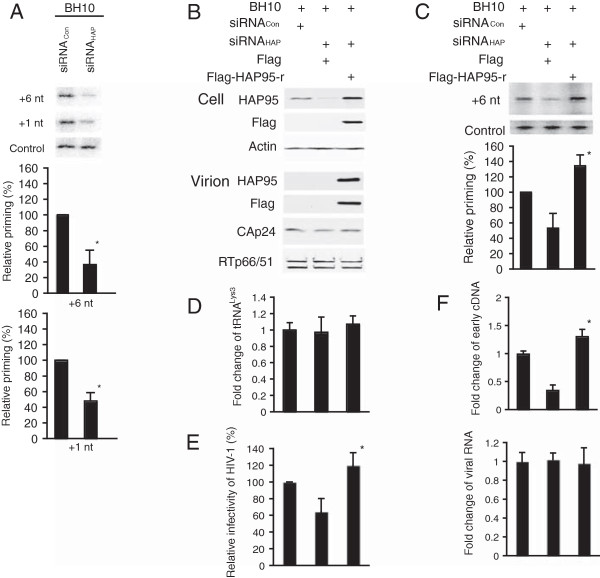
**Decreased cellular HAP95 results in reduced annealing of tRNA** ^**Lys3**^ **to viral RNA. (A)** Total viral RNA was isolated from purified HIV-1 particles, and tRNA^Lys3^ annealed to viral RNA in the cell was extended using reverse transcriptase by either 6 nucleotides (+6 nt) or 1 nt (+1 nt). Upper panel: The extended tRNA^Lys3^ products are resolved by 1D PAGE. The control gel shows that equal amounts of viral RNA were used in each extension reaction. Lower panel: The relative amounts of +6 or +1 nt extended tRNA^Lys3^ products are represented graphically. Shown are the mean values ± SD of 3 experiments. *, *P* < 0.05. **(B)** Rescue of the annealing of tRNA^Lys3^ inhibited by siRNA_HAP_ by expression of HAP95 mRNA resistant to siRNA_HAP_. Upper panel: Western blots of cellular lysates probed with anti-HAP95, anti-Flag, or anti-β-actin. Lower panel: Western blots of viral lysates containing equal amounts of CAp24. **(C)** +6 nt extension assay. Upper panel: +6 nt-extended tRNA^Lys3^ detected in 1D PAGE. Lower panel: The relative amounts of +6 nt extended tRNA^Lys3^ products are represented graphically. *, *P* < 0.05 compared to the results obtained with virions produced from the cells expressing Flag tag only. **(D)** Quantitation of tRNA^Lys3^ in HIV-1 particles by real-time RT-PCR. Shown are the averages of fold change ± SD of 3 experiments. **(E)** Viral infectivity assay was performed as described in Figure 2 legend. *, *P* < 0.05 compared to the results obtained with virions produced from the cells expressing Flag tag only. **(F)** HIV-1 cDNA synthesis in the newly BH10-infected SupT1 cells. The amounts of viral RNA or newly synthesized cDNA in the cells were quantitated by real-time RT-PCR or real-time PCR. *, *P* < 0.05 compared to the results obtained with virions produced from the cells expressing Flag tag only.

Figure 4A (upper panel) shows the 1D PAGE patterns of both +6 and +1 nt extension products using viral RNA. Equal amounts of viral genomic RNA, determined by dot blot hybridization, were used in these reactions, and this was further tested for with a control (control panel) that involves annealing a DNA primer complementary to viral RNA sequences downstream of the PBS and extending it by +6 nt. The results were quantitated, normalized to results obtained with siRNA_Con_, and shown graphically in lower panel. Knockdown of endogenous HAP95 results in reductions in both +6 nt and +1 nt extension of tRNA^Lys3^. The ability of exogenous HAP95 to rescue the reduced annealing of tRNA^Lys3^ due to reduced endogenous HAP95 was examined next. 293T cells were first treated with siRNA_Con_ or siRNA_HAP_, and 16 hours later, were cotransfected with SVC21.BH10 and a vector coding either Flag tag only or Flag-HAP95-r. Flag-HAP95-r has the amino acid sequence identical to that of Flag-HAP95, but the encoding mRNA lacks the siRNA_HAP_ target sequence. The Western blots of cell and viral lysates (Figure 4B) show that the reduction in endogenous HAP95 by siRNA_HAP_ is rescued by Flag-HAP95-r. This exogenous HAP95 results in a detectable presence of this molecule in the virion. The viral content of RTp66/51, relative to CAp24, remains unaffected. As a result of the expression of exogenous HAP95, the tRNA^Lys3^ annealing (Figure 4C) and the viral infectivity (Figure 4E) that were reduced by siRNA_HAP_ are now increased over that obtained in the presence of siRNA_Con_ although the packaging of tRNA^Lys3^ into virus particles remains unchanged (Figure 4D). We further examined the *in vivo* reverse transcription activity of tRNA^Lys3^-viral RNA complex by measuring the level of newly synthesized ssscDNA using real-time PCR after the virus infection of SupT1 cells (Figure 4F). The amounts of viral RNA were determined by real-time RT-PCR as a control. The results show that the virus particles produced from cells containing decreased HAP95 generated significantly less ssscDNAs compared with that of viruses produced from siRNA_Con_-treated cells or Flag-HAP95-r-expressing cells. These results implicate an important role of HAP95 in the annealing of tRNA^Lys3^.

### Effect of knockdown of both RHA and HAP95 upon annealing of tRNA^Lys3^ to HIV-1 RNA

**Figure 5:**
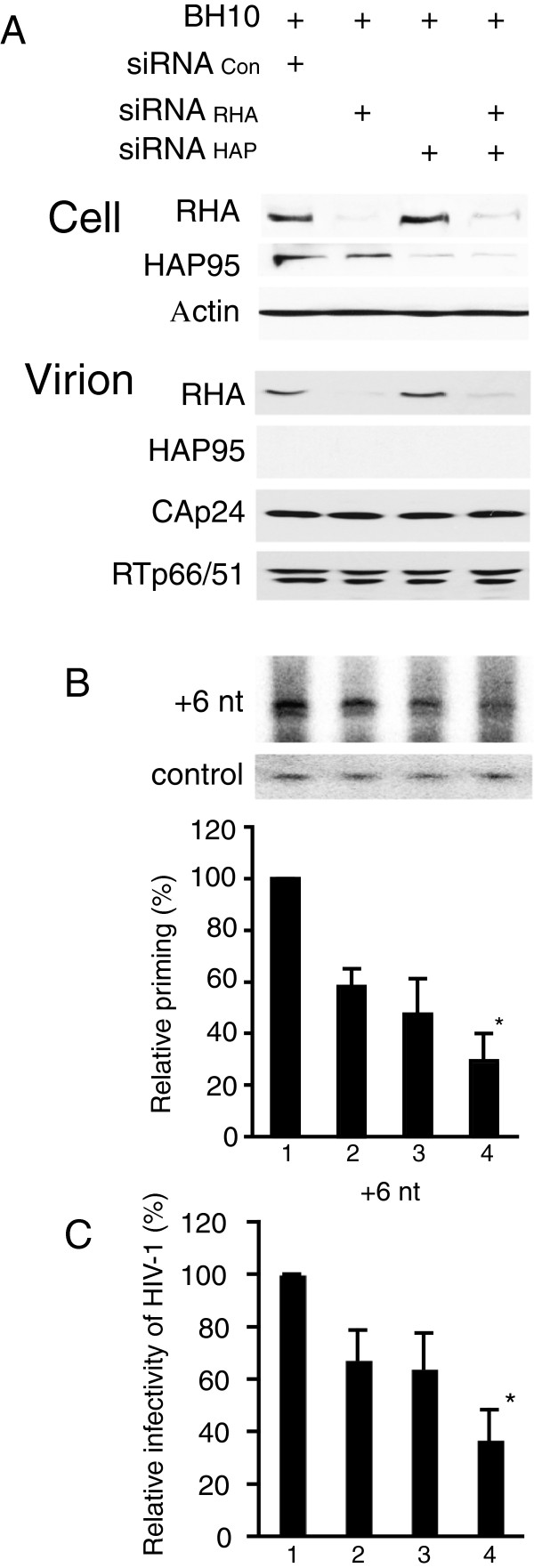
**Effect of knockdown of RHA and HAP95 together upon the annealing of tRNA** ^**Lys3**^ **to viral RNA.** 293 T cells were first treated with siRNA_Con_, siRNA_RHA_, or siRNA_HAP_, or with both siRNA_RHA_ and siRNA_HAP_, and 16 hours later, were transfected with DNA coding for HIV-1 BH10. **(A)** Western blots of cell or viral lysates were probed with antibodies against RHA, HAP95, β-actin, CAp24, or RTp66/p51. **(B)** +6 nt extension assay was performed as described in Figure 4 legend and the results were presented graphically in lower panel. Shown are the mean values ± SD of 3 experiments. **(C)** Viral infectivity assay was performed as described in Figure 2 legend. *, *P* <0.05 compared to values obtained with virions produced from the cells treated with siRNA_Con_, siRNA_RHA_ or siRNA_HAP_ alone (lane 1, 2, or 3 respectively).

Since HAP95 is an RHA binding protein [22, 23] and knockdown of either RHA [14] or HAP95 (Figure 4) reduces the annealing of tRNA^Lys3^ to viral RNA, we studied the effects of reducing both RHA and HAP95 upon the annealing of tRNA^Lys3^ to viral RNA. 293T cells were treated with different siRNAs, and 16 hours later, were transfected with SVC21. BH10. Figure 5 shows the effects of siRNAs to RHA or HAP95, separately or together, upon +6 nt extension of tRNA^Lys3^ by reverse transcription. Figure 5A shows the levels of proteins in the cell or in the virion. The blots of cellular lysate indicate the expected decrease in expression of either RHA or HAP95 in the presence of either siRNA_RHA_ or siRNA_HAP_ respectively. Similarly, in the viral lysate, RHA was reduced in the presence of siRNA_RHA_ alone or together with siRNA_HAP_.

The effects of decreasing endogenous RHA or HAP95, or RHA and HAP95 together, upon +6 nt extension of tRNA^Lys3^ (Figure 5B) and viral infectivity (Figure 5C) were measured. Quantitative values are presented graphically. These results show that reducing both HAP95 and RHA has a greater inhibitory effect on either the annealing of tRNA^Lys3^ or viral infectivity than reducing either one.

### HAP95 inhibits activity of RHA *in vitro*

**Figure 6:**
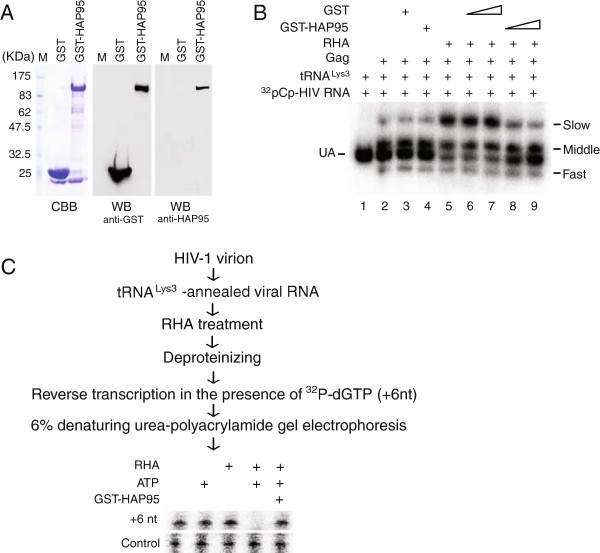
**HAP95 inhibits activity of RHA** ***in vitro*** **. (A)** Purification of GST-tagged HAP95. CBB, The proteins in SDS-PAGE were stained with Commassie brilliant blue R250. WB, Western blots of proteins purified from 293E cells were probed with anti-GST or anti-HAP95. M, protein size marker shown in kDa. **(B)** Annealing of tRNA^Lys3^ to viral RNA *in vitro*. 100 fmoles of tRNA^Lys3^ were annealed to 30 fmoles of ^32^pCp-labeled synthetic viral RNA by Gag in the presence of RHA, GST-HAP95, or both RHA and GST-HAP95. Purified GST was used in place of GST-HAP95 as a negative control. The annealing reaction mixture was incubated at 25°C for 40 min, separated in 5% native polyacrylamide gel, and visualized using a PhosphorImager instrument. Unannealed (UA) viral RNA and the slow-, middle-, and fast-migrating tRNA^Lys3^-annealed viral RNA bands are indicated. **(C)** RHA disrupts tRNA^Lys3^-viral RNA complex isolated from HIV-1 virion. tRNA^Lys3^-annealed viral RNA was isolated from HIV-1 particles, exposed to RHA in the presence or absence of ATP and/or GST-HAP95, extended by reverse transcription in the presence of ^32^P-dGTP, and separated in denaturing 6% polyacrylamide gel. The control panel is the same as described in Figure 4 legend.

RHA promotes the Gag-mediated annealing of tRNA^Lys3^ to viral RNA both *in vivo* and *in vitro* [14]. To study the effect of HAP95 upon the RHA-induced promotion of tRNA^Lys3^ annealing *in vitro,* a 386 nts synthetic HIV-1 RNA was 3’-end labeled with ^32^pCp. GST-tagged HAP95 was purified from 293E cells, as shown by Western blot analysis (Figure 6A). The *in vitro* tRNA^Lys3^ annealing assay was performed using the purified proteins and the reaction mixture was resolved in native 1D PAGE (Figure 6B). When annealing is facilitated with Gag *in vitro*, three tRNA^Lys3^-viral RNA complex bands of different electrophoretic mobility (slow, middle, and fast) were detected, with the middle band being the dominant species [14] (Figure 6B, lane 2). These bands represent tRNA^Lys3^ bound to the PBS of viral RNA [14]. In Figure 6B, we examined the effect of adding RHA to the annealing reaction in the presence of GST or GST-HAP95. All reactions contain the ATP required for RHA activity. RHA facilitates the annealing of tRNA^Lys3^ to viral RNA by increasing the amount of the slow migrating tRNA^Lys3^-viral RNA complex [14] (Figure 6B, lane 5). This increase was compromised by the presence of GST-HAP95 (Figure 6B, lanes 8 and 9), but not GST tag alone (Figure 6B, lanes 6 and 7). However, GST-HAP95 did not affect the formation of middle migrating bands (Figure 6B, lane 4), indicating that HAP95 may inhibit the activity of RHA, but not Gag.

Additionally, a recent report shows that the annealing of tRNA^Lys3^ to the sequence within the primer binding site of HIV-1 facilitates the dimerization of viral RNA [26]. This observation increases the possibility that the slow migrating bands shown in Figure 6B would be a dimerized tRNA^Lys3^-annealed viral RNA. Thus, the identities of the tRNA^Lys3^-viral RNA complexes formed in *in vitro* assay has yet to be further determined.

## Discussion

In this work, we studied the role of HAP95, a binding partner of RHA, in the production of HIV-1. The interest in this protein arises from previous reports showing that RHA promotes annealing of tRNA^Lys3^ to viral RNA [14, 15] and the observation that HAP95 associates with the RT region of HIV-1 Pol in the cell and upon over-expression, is incorporated into HIV-1 particles (Figure 1). Knockdown of HAP95 from cells does not affect the production of virus particles and the packaging of viral RNA or cellular tRNA^Lys3^, a primer for reverse transcription, into virus particles, but results in the reduced amounts of tRNA^Lys3^ annealed on the viral RNA. The impaired tRNA^Lys3^ annealing by decreasing cellular HAP95 was further deteriorated by knockdown of RHA, suggesting a potential cooperation between those two proteins in the cell.

The cooperation between HAP95 and RHA was initially described for simian type D retrovirus replication in which HAP95 binds to RHA and synergizes significantly with RHA to activate CTE-mediated gene expression and promotes nuclear export of unspliced mRNA [22, 23]. However, the cooperative effects between HAP95 and RHA on tRNA^Lys3^ annealing found in this report seem ‘unconventional’ since the HAP95 inhibits the activity of RHA *in vitro* (Figure 6).

**Figure 7:**
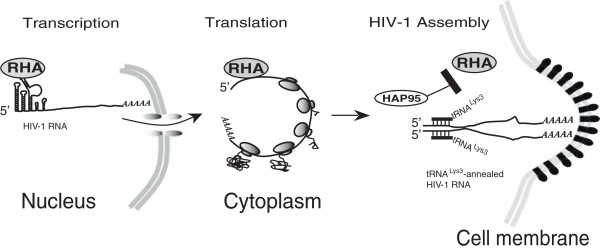
**Model summarizing the involvement of RHA in multiple steps of HIV-1 RNA metabolism and the proposed role of HAP95 in the annealing of tRNA** ^**Lys3**^ **to viral RNA.** RHA mainly associates with the structured 5’-UTR of HIV-1 RNA during virus production. RHA stimulates the accumulation of viral RNA in the nucleus and then by remodeling the highly structured 5’-UTR to facilitate both the viral translation and the annealing of tRNA^Lys3^ to viral RNA in the cytoplasm, and finally assembles into progeny virions. HAP95 is likely to be employed to transiently block the activity of RHA to protect the annealed tRNA^Lys3^ on viral RNA in the cell from being removed by RHA’s activity prior to the budding of virus particles.

Several pieces of data favor a model in which an initial annealing of tRNA^Lys3^ by Gag occurs in the cytoplasm prior to viral budding and protein processing, followed by a re-annealing of the tRNA^Lys3^ by NCp7 after protein processing [9]. Annealing of tRNA^Lys3^ is also facilitated by Gag *in vitro* [11]. Recently, a highly abundant small 18-nt RNA that is antisense to the PBS of HIV-1 was detected in the cells in which HIV-1 was replicating [27]. This 18-nt RNA apparently was generated by cellular RNAi machinery from the double-stranded RNA hybrid formed by the HIV-1 PBS and the 3’ end of the human cellular tRNA^Lys3^, therefore providing direct evidence for the annealing of tRNA^Lys3^ to HIV-1 RNA in the cytoplasm. Since RHA accompanies HIV-1 RNA from transcription in the nucleus until being assembled into progeny virion (Summarized in Figure 7), the double-stranded RNA regions formed in the cytoplasm by annealing of tRNA^Lys3^ to HIV-1 RNA would also be subjected to the activity of RHA, which could remove the annealed tRNA^Lys3^ from viral RNA by unwinding double-stranded RNA region. To support this hypothesis, we have examined the ability of RHA to remove tRNA^Lys3^ that has been annealed to the viral RNA in the cells (Figure 6C). tRNA^Lys3^-annealed viral RNA was isolated from HIV-1 virion produced in non-siRNA-treated 293T cells, and then exposed to enzymatically active RHA at 37°C for 30 min in the presence or absence of 1 mM ATP or GST-HAP95. The viral RNA was then deproteinized and the +6 nt extension assay was performed in an *in vitro* reverse transcription reaction containing ^32^P-dGTP. The extended tRNA^Lys3^ became radioactive and was resolved in denaturing 6% polyacrylamide gels. Control panel of Figure 6C shows the +6 nt extension of a DNA primer that was annealed to viral RNA *in vitro* to verify the presence of viral RNA in the reverse transcription mixtures. The radioactive tRNA^Lys3^ was no longer detectable after RHA treatment in the presence of ATP, indicating that RHA is able to disrupt the tRNA^Lys3^- viral RNA complex that formed in the cell. However, the presence of GST-HAP95 blocked this destructive effect of RHA. The results suggest that it would be necessary to regulate RHA’s activity during HIV-1 production.

RHA has been shown to participate in multiple steps of HIV-1 RNA metabolism [28]. RHA mainly associates with the structured 5’-UTR of HIV-1 RNA during virus production [29]. RHA stimulates the accumulation of viral RNA in the cell [30] and facilitates the generation of unspliced viral RNA [31]. In the cytoplasm, RHA may remodel the structured 5’-UTR of HIV-1 RNA to enhance the translation of viral RNA [32, 33] and also render the viral RNA susceptible to be annealed by tRNA^Lys3^ [14]. According to the observations in this study, it would be plausible to propose that HAP95 may inhibit temporarily the activity of RHA after the placement of tRNA^Lys3^ on the viral RNA prior to viral budding (Figure 7), as HAP95 is able to traffic to the cellular site for HIV-1 particle assembly by association with Pol protein (Figure 1).

## Conclusion

The results support a hypothesis that HAP95 may transiently block RHA’s activity to protect the annealed tRNA^Lys3^ on viral RNA in the cells from removing by RHA during the packaging of RHA into virus particles, thus facilitating the annealing of tRNA^Lys3^ to HIV-1 RNA.

## Methods

### Plasmids

SVC21.BH10 is a simian virus 40-based vector that contains full-length wild-type HIV-1 BH10 proviral DNA [34]. Plasmid hGag/Gag-Pol is a gift from Drs. Y. Huang and G. Nabel at the Vaccine Research Center, National Institute of Allergy and Infectious Diseases (NIAID), National Institutes of Health (NIH), Bethesda, MD [35]. The Gag and Gag-Pol proteins encoded by this plasmid have amino acid sequences identical to that of HIV-1 strain NL4-3, but the mRNA sequences have their codons optimized for efficient translation in mammalian cell. The DNA fragment encoding Pol was amplified from hGag/Gag-Pol by PCR using primers (forward, 5’-ACTAGTGAATTCAGGGAAGATCTGGCCTTCCCA-3’; reverse, 5’-CGATCGGATATCCTAGTCCTCGTCCTGGCGGCT-3’), digested by EcoRI and EcoRV, and then inserted into pcDNA3-N-TAP (provided by Drs. Nahum Sonenberg and Anne-Claude Gingras, McGill university) to yield pcDNA3-N-TAP-hPol expressing TAP-tagged Pol. DNA fragments encoding reverse transcriptase (RTp66) or integrase (INp32) were amplified from hGag/Gag-Pol by PCR using primer pair p66-F/R (5’-CGGGCGGCCGCCCCCATCAGCCCCATCGAG-3’ , forward; 5’-CGGGGCGCGCCCTACAGCACCTTGCGGATGCC-3’ , reverse) or p32-F/R (5’-CGGGCGGCCGCCTTCCTGGACGGCATCGAC-3’ , forward; 5’-CGGGGCGCGCCCTAGTCCTCGTCCTGGCGGCT-3’ , reverse), digested by NotI and AscI, and then inserted into plasmid pN-His to yield pN-His-RTp66 or pN-His-INp32 that expresses 6 × His-tagged RTp66 or INp32 respectively. HAP95 cDNA was amplified by RT-PCR from 293T cells using primer pair (5’-CGGGCGGCCGCCAGCTACACAGGCTTTGTC-3’ , forward; 5’-CGGGGCGCGCCTCATCACGGGGCGCCCCCGCC-3’ , reverse), digested by NotI and AscI, and then inserted into pcDNA3.1-Flag or pTT5SGST to construct pcDNA3.1-Flag-HAP95 or pTT5SGST-HAP95 encoding N-terminally Flag-tagged or GST-tagged HAP95. pcDNA3.1-Flag-HAP95 was mutated by fusion PCR using fusion primers hap-mu-F (5’-CGAATTACGGATACGGGATGGCCACTTCACACTCTT-3’) and hap-mu-R (5’-ATCCCGTATCCGTAATTCGTGGTGTTATCCTGGCCA-3’) to yield pcDNA3.1-Flag-HAP95-r that produces mRNA resistant to siRNA_HAP_, but encodes the identical amino acid residues as pcDNA3.1-Flag-HAP95. Plasmids pcDNA3.1Gag-CTE and pcDNA3.1Gag/Gag-Pol-CTE (provided by Dr. Min Wei, National center for AIDS/STD control and prevention, Chinese center for disease control and prevention, Beijing, China) encoding HIV-1 BH10 Gag or Gag plus Gag-Pol were used to produce HIV-1 VLP by transfection into 293T cells. The extracellular VLPs were purified by step sucrose centrifugation.

### Cell culture

SupT1 cells were cultured in RPMI 1640 medium supplemented with 10% fetal bovine serum (FBS), 1% penicillin-streptomycin, and 1% glutamine and were infected with HIV-1 produced from 293T cells. TZM-bl is a HeLa cell line expressing CD4 and CCR5 and containing a reporter luciferase gene driven by HIV-1 long terminal repeat promoter [36]. This cell line was used for measuring HIV-1 infectivity and was obtained from NIH AIDS Research and Reference Reagent Program, Division of AIDS, NIAID, NIH. 293T and TZM-b1 cells were grown in complete Dulbecco’s modified Eagle’s medium (DMEM) supplemented with penicillin, streptomycin, and 10% FBS. Transfection of 293T cells was done using Lipofectamine 2000 (Invitrogen). HEK 293E cell is a stably transfected HEK293 cell line expressing Epstein-Barr virus nuclear antigen-1 and supports amplification of plasmids containing the replication origin region (OriP) of Epstein-Barr virus, resulting in high expression of proteins encoded for by these plasmids [37]. This cell line was obtained from Mr. Yves Durocher (Biotechnology Research Institute, Montreal, Canada) and was used to express and purify GST-tagged HAP95, grown in F17 medium (Invitrogen) supplemented with 2 mM l-glutamine and 0.1% Pluronic F-68 (GIBCO), and transfected by 25 kDa linear Polyethylenimine (PEI, pH7.0) (Polysciences Inc).

To establish a cell line stably expressing TAP-Pol, 293T cells were transfected with pcDNA3-N-TAP-hPol and then selected for by culturing in medium containing G418. Established cell lines were confirmed by Western blot analysis using rabbit polyclonal antibody against HIV-1 RT. A cell line expressing only TAP tag was established as well by transfecting 293T cells with plasmid pcDNA3-N-TAP and used as a control in protein complex purification.

### Purification and identification of cellular proteins bound to TAP-Pol

We followed the TAP procedure to purify TAP-Pol complex as described [15]. Briefly, 9 × 10^8^ 293T cells stably-transfected with pcDNA3-N-TAP-hPol or pcDNA3-N-TAP were collected, washed with ice-cold phosphate-buffered saline, and then lysed in buffer containing 50 mM HEPES (pH 8.0), 100 mM KCl, 2 mM EDTA, 0.1% Nonidet P-40, 2 mM dithiothreitol, 10 mM NaF, 0.25 mM NaOVO_3_, 5 nM Okadaic acid, 5 nM calyculin A, 50 mM glycerolphosphate, 10% glycerol, and protease inhibitors (Roche Diagnostics). After centrifugation at 19,800 × *g* for 30 minutes, supernatants were incubated with IgG beads (Amersham Biosciences) at 4°C overnight. The TAP-Pol-bound IgG beads were washed with lysis buffer and TEV buffer (10 mM HEPES, pH 8.0, 150 mM NaCl, 0.1% Nonidet P-40, 0.5 mM EDTA, and 1 mM dithiothreitol) and then incubated with TEV protease (Invitrogen) in TEV buffer at 16°C for 2 h to release TAP-Pol proteins. The supernatants were further incubated with calmodulin beads (Amersham Biosciences) in calmodulin-binding buffer (10 mM HEPES, pH 8.0, 150 mM NaCl, 1 mM MgOAc, 1 mM imidazole, 0.1% Nonidet P-40, 2 mM CaCl_2_, and 10 mM β-mercaptoethanol). After washing with calmodulin-binding buffer and calmodulin rinsing buffer (100 mM HEPES, pH 8.0, 75 mM NaCl, 1 mM MgOAc, 1 mM imidazole, and 2 mM CaCl_2_), TAP-Pol protein complex was eluted by using a buffer containing 200 mM HEPES, pH 8.0, 75 mM NaCl, 1 mM MgOAc, 1 mM imidazole, and 25 mM EGTA. The eluted proteins were separated in 10% SDS-PAGE followed by Coomassie Brilliant Blue R250 staining. The visible protein bands were selectively excised, in-gel-digested with trypsin, and subjected to liquid chromatography/mass spectrometry analysis (Calgary University Sams Centre).

### Viral RNA isolation

The amounts of HIV-1 particles were determined by measuring the level of CAp24 antigen using the enzyme-linked immunosorbent assay (ELISA). The extracellular HIV-1 particles were purified by step sucrose centrifugation. The viral RNA was isolated from purified virus particles using guanidinium isothiocyanate as described [38] and dissolved in 5 mM Tris buffer (pH 7.5).

### Packaging of tRNA^Lys3^ and viral RNA in HIV-1 particles

The abundance of tRNA^Lys3^ or HIV-1 genomic RNA in virus particles was determined by dot blot hybridization as described [39]. Briefly, each sample of total viral RNA isolated from equal amounts of purified virus particles was blotted onto Hybond N + nylon membranes (Amersham Pharmacia) and probed with a ^32^P-labeled 18-mer DNA specific for the 3’ end of tRNA^Lys3^ (5’-TGGCGCCCGAACAGGGAC-3’) or ^32^P-labeled DNA oligonucleotides that are complementary to 3 different regions of HIV-1 genomic RNA (p2830, 5’-AGGCTGTACTGTCCATTTATCAGGATGGAGT-3’; p5671, 5’-TATTGCTATTATTATTGCTACTACTAATGCT-3’; p8304, 5’-TGTGGCGAATAGCTCTATAAGCTCCTT-3’). The radioactive signals were detected and quantitated using a PhosphorImager instrument (Amersham Pharmacia).

### Primer extension assay

tRNA^Lys3^-primed initiation of reverse transcription was analyzed by measuring the ability of tRNA^Lys3^ to be extended by one nucleotide (+1 nt) or six nucleotides (+6 nt) in an *in vitro* reverse transcription reaction. The amounts of total viral RNA used in the reactions were first quantitated by dot blot hybridization as described above and were further determined by measuring the ability of a DNA primer (5’-TCTAATTCTCCCCCGCTTAATACTGACGCT-3’) annealed at room temperature to the viral RNA to prime a +6 nt extension (5’-CTCGCA-3’). Equal amounts of total viral RNA were used as the source of primer tRNA^Lys3^ annealed *in vivo* to viral genomic RNA. The sequence of the first six deoxynucleoside triphosphates incorporated is 5’-CTGCTA-3’. For +6 nt extension, reactions were carried out in a volume of 20 μl containing 50 mM Tris–HCl (pH 7.8), 100 mM KCl, 10 mM MgCl_2_, 10 mM dithiothreitol, 0.2 mM dCTP, 0.2 mM dTTP, 5 μCi of α-^32^P-dGTP (0.16 μM), and 0.05 mM ddATP (instead of dATP, thereby terminating the reaction at the sixth base), 50 ng of HIV-1 RT, and RNase inhibitor (Ambion). For +1 nt extension, reactions were carried out in a volume of 20 μl RT reaction mixture containing only 0.16 μM α-^32^P-dCTP. Reaction mixture was incubated at 37°C for 15 min. The extended tRNA^Lys3^ was precipitated with isopropanol and separated in a 6% denaturing polyacrylamide gel.

### Real-time PCR

Real-time PCR was performed using PerfeCta SYBR green FastMix (Quanta BioScience Inc.) as instructed by the manufacturer’s instructions. The PCR conditions were 95°C for 5 s, 60°C for 15 s, and 72°C for 10 s. All analyses were done in triplicate, with triplicate samples in each experiment.

### Quantitation of newly synthesized HIV-1 cDNA, viral genomic RNA, and tRNA^Lys3^

Real-time PCR was carried out to examine the synthesis of viral cDNA intermediates in a permissive cell line SupT1 that has been infected with HIV-1 BH10 produced from siRNA–treated 293T cells. 3 × 10^6^ SupT1 cells were infected with equal amounts of purified virions (100 ng of CAp24) at 4°C for 2 h. The cells were then washed twice with phosphate-buffered saline, and incubated at 37°C. At 16 hours post-infection, the cells were collected and washed with phosphate-buffered saline. The cellular DNA was extracted using the DNeasy tissue kit (Qiagen). Equal amounts of cellular genomic DNA were used in real-time PCR to quantitate viral cDNA intermediates containing sequence for R-U5 using the following primer set: 5’-TTAGACCAGATCTGAGCCTGGGAG-3’/5’-GGGTCTGAGGGATCTCTAGTTACC-3’ [40]. Total cellular RNA was also isolated from HIV-1-infected SupT1 cells using TRIzol (Invitrogen). The amount of β-actin mRNA was quantitated using real-time RT-PCR as described [41] as an internal control. The total cellular RNA containing equal amount of β-actin mRNA was used to generate cDNA of the viral genomic RNA using SuperScript reverse transcriptase III (Invitrogen) and the primer (5’-AGCCTTCTCTTCTACTACTTTTACCC-3’). The amount of resultant cDNA was determined by real-time PCR using the following primer set: 5’-CTACAACCATCCCTTCAGACAGGAT-3’/5’-TCCTGTGTCAGCTGCTGCTTGCTGT-3’. These primers were also used in real-time RT-PCR to quantitate viral RNA isolated from equal amounts of HIV-1 virions. The amount of tRNA^Lys3^ in the same viral RNA sample was quantitated by real-time RT-PCR using following primer set: 5’-GTCGGTAGAGCATCAGACTT-3’/5’-CGCCCGAACAGGGACTT-3’ [42].

### Virus infectivity assay

Single-cycle infectivity assay was performed as described [14]. 10^5^ TZM-bl cells were challenged with viruses corresponding to 5 ng of CAp24. The induced activity of luciferase in cell lysates was measured using luminometer Lumat LB9507 (EG&G Berthold, Bad Wildbad, Germany).

### Isolation of HIV-1 core

HIV-1 core was isolated by following the procedures described [43]. Briefly, culture medium was clarified by centrifugation and then virus particles were concentrated through a step cushion of sucrose (15% and 65%, wt/wt) in phosphate-buffered saline. The virus pellet was slowly resuspended in phosphate-buffered saline and then mixed with an equal volume of 200 mM NaCl-100 mM morpholinepropanesulfonic acid (MOPS, pH 7.0). Triton X-100 was added to a final concentration of 0.5% to lyse virions for 2 min at room temperature. HIV-1 cores were recovered by centrifugation in a microcentrifuge at full speed (13,400 g) for 8 min at 4°C, washed twice with 100 mM NaCl-50 mM MOPS (pH 7.0), resuspended in the same buffer, and analyzed immediately by Western blotting.

### siRNA

Small interfering RNA (siRNA) against RHA has been described [14]. siRNAs against HAP95 (siRNA_HAP_) were generated using the target sequence 5’-CCAACUAUGGGUAUGGUAU-3’, and purchased from Invitrogen. Lipofectamine 2000 (Invitrogen) was used to introduce siRNAs into 293T cells. The endogenous RHA and HAP95 were measured by Western blot analysis using specific antibodies. The level of β-actin was measured as an internal control.

### Purification of proteins

Purification of RHA and HIV-1 Gag-pr55 has been described [14]. The procedures developed by Durocher et al. [44] were followed for purifying glutathione-s-transferase (GST) or GST-tagged HAP95 from 293E cells. Briefly, the cells were transfected with pTT5-SGST or pTT5-SGST-HAP95. The transfected cells were collected at 48 hours later, washed with ice-cold phosphate-buffered saline, and then lysed in buffer containing 50 mM NaH_2_PO_4_, 300 mM NaCl, 0.5% Triton-X 100, 10% glycerol, protease inhibitor cocktail tablets (Roche), pH7.4. The cell lysates were clarified by centrifugation at 19,800 g for 30 min at 4°C. Glutathione Sepharose high performance (GE healthcare) was added into supernatant and incubated at 4°C for 2 hours to capture GST or GST-HAP95. After extensive washes, the protein was eluted by 33 mM reduced glutathione (Fisher Scientific) in elution buffer containing 50 mM Tris–HCl, pH8.0 and 10% glycerol. Purified proteins were dialyzed against 20 mM Tris–HCl, pH7.5, 150 mM NaCl, 20 mM KCl, 2 mM MgCl_2_, 2 mM dithiothreitol, 10% glycerol, and then stored at -80°C.

### *In vitro*tRNA^Lys3^annealing assay

The first 386 nts of 5’-UTR of HIV-1 RNA and tRNA^Lys3^ were synthesized by *in vitro* transcription using T7 RNA polymerase (T7 MEGAscript kit, Ambion) [14]. Synthetic viral RNA was labeled with [5’-^32^P] Cytidine 3’,5’-bis (phosphate) (^32^pCp) using T4 RNA ligase (Fermentas). 30 fmoles of ^32^pCp-labeled viral RNA and 100 fmoles of tRNA^Lys3^ were first incubated with 1,000 fmoles of RHA at 37°C for 10 minutes in the presence of 3,000 or 6,000 fmoles of GST or GST-tagged HAP95 in 20 μl reaction mixture containing 10 mM Tris–HCl, pH 8.0, 50 mM KCl, 2 mM MgCl_2_, 2 mM dithiothreitol, 2 units RNasin, 2.5 mM NaH_2_PO_4_, 15 mM NaCl, and 2 mM ATP. Gag (20,000 fmoles) was then added and incubated at 25°C for 40 min. The reaction was stopped by addition of 5 μl solution containing 2% SDS, 10 mM CaCl_2_, 250 μg/ml proteinase K, 40% glycerol, bromophenol blue, and xylene cyanol. Twenty minutes later, the samples were electrophoresed on a 5% native polyacrylamide gel in 0.5 × Tris-borate-EDTA buffer (TBE). ^32^pCp-labeled viral RNAs were visualized and quantitated using a PhosphorImager instrument.

### Protein co-precipitation

Protein complexes containing His-tagged protein were precipitated from cellular lysates by using Ni-nitrilotriacetic acid (NTA) agarose (Qiagen). The transfected cells were lysed in lysis buffer (50 mM NaH_2_PO_4_, pH8.0, 150 mM NaCl, 10 mM imidazole, 0.5% Triton-X 100, 10% glycerol, protease inhibitor cocktail tablets) and then centrifuged at 10,000 g for 10 min at 4°C. Ni-NTA agarose was added into the clarified supernatant and incubated at 4°C for 2 hours. Precipitated protein complex was eluted by 250 mM imidazole and analyzed by Western blotting.

### Western blot analysis

The protein samples were resolved in SDS-PAGE and then blotted onto nitrocellulose membranes (Bio-Rad). Western blots were probed with rabbit anti-HIV RT, mouse anti-p24 [45], mouse anti-gp120 [46, 47] (NIH AIDS Research and Reference Reagent Program, Division of AIDS, NIAID, NIH), rabbit anti-HAP95 (Proteintech Group Inc), monoclonal anti-human β-actin (Sigma), polyclonal rabbit anti-RHA (Novus Biologicals), or monoclonal anti-polyHistidine (Sigma). Horseradish peroxidase-conjugated secondary antibodies used were either anti-rabbit immunoglobulin (1:5,000 dilution) (Amersham Pharmacia Biotech) or anti-mouse immunoglobulin (1:5,000 dilution) (Rockland Immunochemicals). Protein bands were detected by enhanced chemiluminescence (ECL, Perkin-Elmer Life Science Inc).

### Statistical analysis

The one tailed Student’s *t* test was employed in statistical analyses. The lowest level of significance was set at *P* < 0.05.

## Abbreviations

HIV-1: Human immunodeficiency virus 1
UTR: Untranslated region
RHA: RNA helicase A
ELISA: Enzyme-linked immunosorbent assay
HAP95: A-kinase anchoring protein 95-like protein
AKAP95: A-kinase anchoring protein 95
NC: Nucleocapsid
RT: Reverse transcriptase
CTE: Cis-acting constitutive transport element
ssscDNA: Minus strand strong stop cDNA
VLP: Virus-like particle
FBS: Fetal bovine serum
TAP: Tandem affinity protein purification system
PBS: Primer binding site
SDS: Sodium dodecyl sulfate
PAGE: Polyacrylamide gel
pCp: [5’-^32^P] Cytidine 3’,5’-bis (phosphate).
